# Clinical Results of the Advanced Neurovascular Access Catheter System Combined With a Stent Retriever in Acute Ischemic Stroke (SOLONDA)

**DOI:** 10.1161/STROKEAHA.121.037577

**Published:** 2022-04-01

**Authors:** Manuel Requena, Marc Ribo, Joaquin Zamarro, Pedro Vega, Jordi Blasco, Eva María González, María Del Mar Freijo, Jose Carlos Mendez Cendón, María Ángeles de Miquel, David Hernández, Manuel Moreu, Sebastià Remollo, Sonia Sánchez, David S. Liebeskind, Tommy Andersson, Christophe Cognard, Raul Nogueira, Alejandro Tomasello

**Affiliations:** Stroke Unit, Department of Neurology (M. Requena, M. Ribo), Hospital Universitari Vall d’Hebron, Barcelona, Spain.; Interventional Neuroradiology Section, Department of Radiology (M. Requena, D.H., A.T.), Hospital Universitari Vall d’Hebron, Barcelona, Spain.; Interventional Neuroradiology, Hospital Universitario Virgen de la Arrixaca, Murcia, Spain (J.Z.).; Interventional Neuroradiology, Department of Radiology, Hospital Universitario Central de Asturias, Spain (P.V.).; Department of Radiology, Hospital Clínic, Barcelona, Spain (J.B.).; Interventional Neuroradiology, Department of Radiology (E.M.G.), Hospital Cruces, Bilbao, Spain.; Department of Neurology (M.D.M.F.), Hospital Cruces, Bilbao, Spain.; Department of Radiology, Ramón y Cajal University Hospital, Madrid, Spain (J.C.M.C.).; Department of Radiology, Hospital Universitari de Bellvitge, Barcelona, Spain (M.A.d.M.).; Neurointerventional Radiology, Department of Radiology, Hospital Clínico San Carlos, Madrid, Spain (M.M.).; Neurointerventional Radiology Unit, Department of Neurosciences, Hospital Germans Trias i Pujol, Badalona, Spain (S.R.).; Anaconda Biomed, Barcelona, St Cugat del Valles, Spain (S.S.).; Neurovascular Imaging Research Core and University of California Los Angeles Stroke Center, Department of Neurology, University of California (D.S.L.).; Department of Medical Imaging, Groeninge Hospital, Kortrijk, Belgium (T.A.).; Departments of Neuroradiology, Karolinska University Hospital and Clinical Neuroscience, Karolinska Institute, Stockholm, Sweden (T.A.).; Hôpital Purpan, Diagnostic and Therapeutic Neuroradiology, Toulouse, France (C.C.).; Department of Neurology, Marcus Stroke and Neuroscience Center, Grady Memorial Hospital and Department of Neurology, Atlanta, GA (R.N.).

**Keywords:** catheters, ischemic stroke, reperfusion, stents, thrombectomy

## Abstract

**Background:**

The Advanced Neurovascular Access (ANA) thrombectomy system is a novel stroke thrombectomy device comprising a self-expanding funnel designed to reduce clot fragmentation by locally restricting flow while becoming as wide as the lodging artery. Once deployed, the ANA device allows distal aspiration combined with a stent retriever to mobilize the clot into the funnel where it remains copped during extraction. We investigated the safety and efficacy of ANA catheter system.

**Methods:**

SOLONDA (Solitaire in Combination With the ANA Catheter System as Manufactured by Anaconda) was a prospective, open, single-arm, multicenter trial with blinded assessment of the primary outcome by an independent core lab. Patients with anterior circulation vessel occlusion admitted within 8 hours from symptom onset were eligible. The primary end point was successful reperfusion (modified Thrombolysis in Cerebral Infarction score 2b–3) with ≤3 passes of the ANA device in combination with stent retriever, before the use of rescue therapy in the intention to treat population. Primary predefined analysis was noninferiority as compared to the performance end point observed in HERMES (High Effective Reperfusion Using Multiple Endovascular Devices).

**Results:**

After enrollment of 74 patients, an interim analysis was conducted, and the trial Steering Committee decided to terminate recruitment due to safety and performance objectives were reached. Mean age was 71.6 (SD 8.9) years, 46.6% women and median National Institutes of Health Stroke Scale on admission 14 (interquartile range, 10–19). Successful reperfusion within 3 passes before rescue therapy was achieved in 60/72 (83.3% [95% CI, 74.7%–91.9%]) with a rate of complete reperfusion (modified Thrombolysis in Cerebral Infarction score 2c–3) of 60% (95% CI, 48.4%–71.1%; 43/72 patients). After noninferiority was confirmed (*P*<0.01), the ANA device also showed superiority in the rate of successful reperfusion with ≤3 passes (*P*=0.02). First-pass successful recanalization rate was 55.6% (95% CI, 44.1%–67.0%), with a first-pass complete recanalization rate of 38.9% (95% CI, 27.6%–50.1%). Rescue therapy to obtain a modified Thrombolysis in Cerebral Infarction score 2b–3 was needed in 12/72 (17%) patients. At 90 days, the rate of favorable functional outcome (modified Rankin Scale score 0–2) was 57.5% (95% CI, 46.2%–68.9%), and the rate of excellent functional outcome (modified Rankin Scale score 0–1) was 45.2% (95% CI, 33.8%–56.6%). The rate of severe adverse device related was 1.4%.

**Conclusions:**

In this clinical experience, the ANA device achieved a high rate of complete recanalization with a preliminary good safety profile and favorable 90 days clinical outcomes.

**Registration:**

URL: https://www.clinicaltrials.gov; Unique identifier: NCT04095767.

Mechanical thrombectomy (MT) has become the standard of care for acute ischemic stroke due to a large vessel occlusion based on the evidence of pivotal clinical trials.^1,2^ Time from onset to reperfusion, and complete reperfusion with a minimum number of attempts are the main modifiable factors associated with improved clinical outcome and the main goal of interventionalists.

In a pooled meta-analysis of major trials successful reperfusion (defined as modified Thrombolysis in Cerebral Ischemia [mTICI] score 2b–3) was observed in 71%^3^ of the patients, and the rate of complete reperfusion (mTICI score 3) in 33%. Moreover, the rate of complete revascularization (mTICI score 2c–3) after a single attempt with MT (first-pass effect) was reported to be 25% to 40%^4^ in larger series of consecutive patients receiving endovascular treatment.

Distal embolization^5^ or clot fragmentation might occur during the procedure leading to incomplete recanalization. Specific device features such as flow arrest induction^6^ or catheter-to-vessel ratio^7^ have been described as predictors of complete recanalization by reducing distal embolization or allowing complete clot ingestion into the catheter without fragmentation.^8^

The Advanced Neurovascular Access (ANA device or ANA catheter system, Anaconda Biomed, Barcelona, Spain) is a new stroke thrombectomy device comprising a self-expanding funnel component designed to reduce side effects generated by the clot fragmentation through locally restricting blood flow while becoming as wide as the diameter of the lodging artery. Once deployed, ANA device allows distal aspiration combined with a stent retriever (SR) to mobilize the clot into the funnel where it remains trapped and protected during extraction. In in vitro phantom and swine models of the human neurovascular anatomy, the ANA device showed better recanalization rates than SR in combination with a distal aspiration catheter or a balloon guiding catheter.^9,10^ A preliminary first in human report indicated promising results in terms of safety and efficacy.^11^

The SOLONDA study (Solitaire in Combination With the ANA Catheter System as Manufactured by Anaconda) aimed to assess the safety and performance of the ANA catheter system combined with a SR in acute ischemic stroke.

## Methods

The data supporting the findings of this study are available from the corresponding author upon reasonable request.

### Study Design

SOLONDA was a prospective, open, single-arm, multicenter study (9 centers in Spain) with blinded assessment of the primary outcome by an independent core lab (study protocol in the Supplemental Material). The investigational use of the ANA device and clinical protocol were approved by the national regulatory agency and by the Institutional ethics committee (June 7, 2019,—study number 5482). Informed consent to participate in the trial was obtained from all patients or next of kin before treatment. Patients did not receive any compensation for participating in this study.

### Description of ANA Device and Procedure

**Figure 1.**
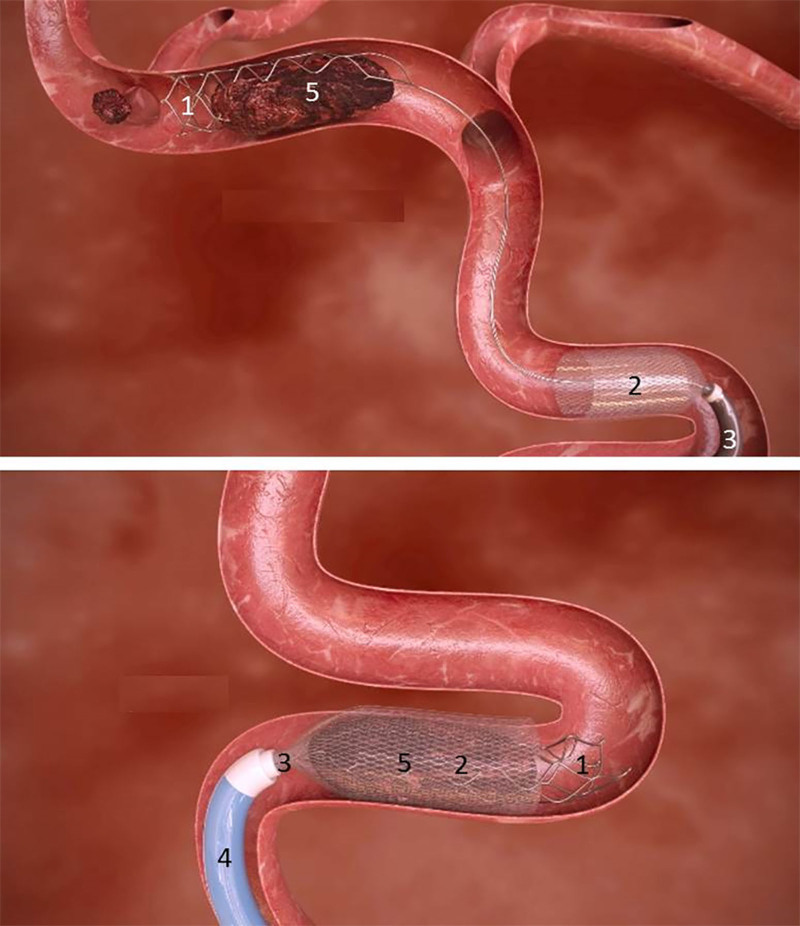
**Schematic drawing of thrombectomy using Advanced Neurovascular Access (ANA) device in combination with a stent retriever.** (1) Solitaire deployed stent retriever, (2) ANA expanded funnel, (3) ANA delivery catheter, (4) guide catheter, and (5) clot.

**Figure 2.**
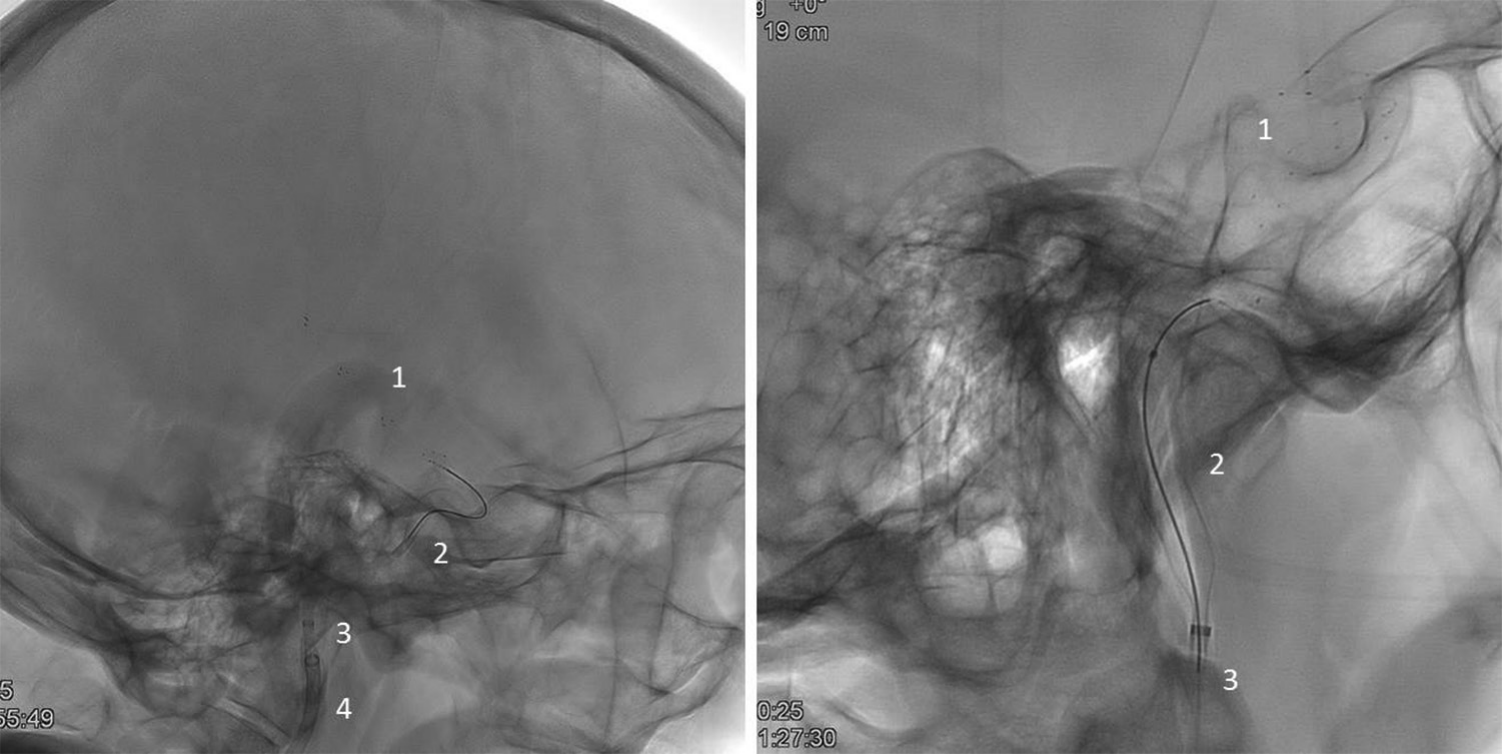
**Representative images of thrombectomy procedure with Advanced Neurovascular Access (ANA) device in combination with Solitaire stent retriever.** Thrombectomy setting with devices deployed. (1) Solitaire deployed stent retriever, (2) ANA expanded funnel, (3) ANA delivery catheter, and (4) guide catheter.

ANA is a thrombectomy device, comprised of 2 coaxial catheters: the funnel catheter and the delivery catheter, made from variable stiffness sections (Figures 1 and 2). The funnel catheter comprises a braided self-expanding metallic structure fully covered with a highly flexible polymer. It is intended to restrict the blood flow locally during the intervention, and it can provide effective aspiration that serves as a complementary mechanism combined with a SR. The delivery catheter is the outer catheter of the device, which navigates until reaching the target vessel. In all patients, once the guide catheter was placed at the level of the internal carotid artery, in a triaxial setting, a microcatheter was advanced over a micro guidewire to the clot. The ANA catheter system, in its retracted position, was then positioned as close as possible to the proximal aspect of the clot (terminal internal carotid artery or middle cerebral artery), and the funnel was deployed to restrict flow locally. The funnel self-expands then up to the diameter of the artery. The microcatheter was then advanced through the occlusion site, and the SR (Solitaire family, Medtronic) deployed as in usual practice. At this point, the microcatheter was withdrawn entirely to increase the aspiration lumen, and manual aspiration was applied through the ANA funnel catheter. When the distal ends of SR and ANA funnel catheter were aligned, the ANA and the SR were simultaneously pulled out. During the extraction through the guiding catheter, the clot was held in place inside the funnel with means of 2 retention mechanisms: the force exerted by the aspiration applied through the funnel, and the compression of the braid under axial stress (Chinese finger trap effect). The expanded funnel confers a substantial increase in suction force compared with other catheters, specifically in combination with a SR that contributes to the efficacy to retrieve the clot.^12^ All participating interventionalists were trained on the use of the ANA device before start of enrollment. The training was performed on a simulation cerebrovascular model where procedural steps including navigation and removal of an artificial clot were practiced.

### Population

Patients aged from 18 to 85 years old with an acute ischemic stroke due to a large artery occlusion in the anterior circulation such as the internal carotid artery and M1 or M2 segments of the middle cerebral artery admitted within the timeframe of 8 hours from symptom onset to groin puncture in the catheterization lab were eligible. Inclusion criteria included National Institutes of Health Stroke Scale (NIHSS) score 8 to 25 at onset, prestroke modified Rankin Scale (mRS) score 0–1, and Alberta Stroke Program Early CT Score >5 (full list of inclusion and exclusion criteria is included in the Supplemental Material). The use of standard-dose thrombolysis was at the discretion of the treating physician following current guidelines.

### Clinical and Radiological Assessments

All patients underwent a clinical assessment, including demographics and medical history, physical exam (blood pressure and heart rate), baseline laboratory tests, 12-lead ECG, NIHSS score, and mRS on admission by certified neurologists to evaluate prestroke disability status. The NIHSS scores were also performed after 24 hours and at 5 days, and the mRS score at 90 days.

Diagnostic neuroimaging (computed tomography or magnetic resonance imaging) was performed on admission and after 24 hours. Radiological variables such as the Alberta Stroke Program Early CT Score, the presence and location of the vessel occlusion and intracranial hemorrhage detection at 24-hour follow-up were assessed. The local interventionalists and the core lab assessed all neuroimaging including the rate of recanalization according to the expanded treatment in cerebral ischemia,^13^ including first-pass complete recanalization (mTICI score 2c–3) and rate of successful recanalization (mTICI score 2b–3) after each pass and at the end of procedure, including any rescue therapy. Intraprocedural imaging adverse events were evaluated by the core lab. An independent Data Safety Monitoring Board monitored the safety of the subjects and the integrity of the generated data. All adverse events were adjudicated by an independent Clinical Event Committee.

Only core lab readings were considered for primary end point analyses and presented as the main results. In 3 cases, local readings were used as images could not be transferred to the central imaging core lab. An independent Clinical Research Organization was responsible for the monitoring and quality of the clinical data (MedPass/ICON, France).

### Outcomes

The primary performance end point of the study was the ability of the ANA device to facilitate stentriever deployment and to perform thrombectomy in the anterior circulation, with successful reperfusion, defined as mTICI score of 2b–3 in the target vessel with ≤3 passes of the investigational device before the use of rescue therapy.

The primary safety end point was the occurrence of all serious adverse device effects (SADE) up to 90-day postprocedure, including symptomatic intracerebral hemorrhage, at 24 hours (−8/+12h) postprocedure.

Secondary performance outcomes included the ability of the investigational device to reach the neurovasculature (passing the bulb of the internal carotid artery in the anterior cerebral circulation) allowing navigation and deployment of the SR to attempt thrombectomy, procedure time, neurological status at day 5 or discharge and functional outcome measured by mRS at 90 days. A favorable outcome was defined as mRS score ≤2.

Secondary safety outcomes included any intracerebral hemorrhage, rate of neurological deterioration (worsening ≥4 points on NIHSS), embolization and infarct in a previously uninvolved vascular territory, procedural complications at 24 hours, and procedure-related mortality at day 5 or discharge.

### Sample Size Determination

To define the number of subjects to be included in the study, a review of the literature available at the time of study design was conducted to define the performance targets to achieve for the primary performance end point. In a meta-analysis of previous stroke trials^3^ (HERMES [High Effective Reperfusion Using Multiple Endovascular Devices], pooling patient-level data from 5 trials: MR CLEAN [Multicenter Randomized Clinical Trial of Endovascular Treatment for Acute Ischemic Stroke in the Netherlands], ESCAPE [Endovascular Treatment for Small Core and Proximal Occlusion Ischemic Stroke], REVASCAT [Endovascular Revascularization With Solitaire Device Versus Best Medical Therapy in Anterior Circulation Stroke Within 8 Hours], SWIFT PRIME [Solitaire With the Intention for Thrombectomy as Primary Endovascular Treatment], and EXTEND IA [Extending the Time for Thrombolysis in Emergency Neurological Deficits - Intra-Arterial], 570 anterior circulation thrombectomy patients, final mTICI results) and on the publication by Garcia-Tornel et al^14^ (Vall d’Hebron Barcelona, 704 anterior circulation thrombectomy patients, mTICI after 3 passes), the combined incidence for the primary performance (mTICI score 2b–3 with ≤3 passes) end point was 71.1% with a 95% binomial CI of (68.5%–73.8%). As a result, the target for the primary end point was set at 71%.

In a noninferiority setting, considering an alpha of 2.5% in a unilateral approach, a noninferiority margin of 2% (which corresponds to the lower bound of the confidence interval reported in the literature), and expecting a success rate for the ANA device of 83%, a sample size of N=110 subjects would provide a power around 90% to the following statistical test for binomial proportion: null hypothesis as success of ANA device ≤69% and alternative hypothesis as success of ANA device >69%.

In a superiority testing, considering an alpha of 5% in a bilateral approach and expecting a success rate for the ANA device of 83%, a sample size of N=110 subjects would provide a power around 80% to the following statistical test for binomial proportion: null hypothesis as success of ANA device of 71% and alternative hypothesis as success of ANA device different than 71%.

Considering possible loss of subjects, and with the intent to provide an informative sample for the safety analysis, the final sample size of the study was increased to N=125 subjects to meet the objectives of this clinical evaluation.

### Statistical Analysis

Statistical analyses were done using SAS System. All statistical analyses were made on locked databases following data clarifications resolved due to data management processes.

The following study populations were defined for the purpose of statistical analysis:

1. Enrolled population: defined as all subjects who have given their informed consent to participate in the study.
2. Intent-to-treat (ITT): defined as all subjects enrolled in the study for whom at least one attempt to introduce the ANA catheter system has been made.
3. Modified ITT (mITT): defined as all subjects from the ITT analysis set, with the exclusion of roll-in subjects. A roll-in subject was defined as the first treated subject for each interventionalist investigator.

Primary analysis set for statistical reporting was the ITT population, with no replacement of missing data planned in the statistical analysis to provide unbiased results. However, 2 sensitivity analyses were conducted regarding the missing values for the primary performance end point. The conservative one imputed failure for the missing values. In addition, primary end points were also reported on the mITT population to assess a potential learning effect regarding use of the device.

Except for the primary performance end point, no statistical testing was conducted for any parameters in the study and only descriptive analysis was provided to fully describe the parameters recorded.

Continuous variables were summarized using standard quantitative statistics: number of nonmissing observations, mean, SD, median, quartiles, and range (minimum and maximum observed values). The number of missing observations were also specified.

Categorical variables were summarized using classical frequency statistics: number of nonmissing observations and percentages by categories. Percentages were calculated on the number of nonmissing observations.

Adverse events data were summarized using descriptive statistics: total number of events and number of subjects with at least one of the respective categories (adverse events, adverse device effects [ADEs], serious adverse events, and SADEs) and device deficiencies. The severity and the causal relationship are presented as adjudicated by the Clinical Event Committee.

### Trial Termination

After enrollment of 74 patients, an interim analysis was conducted. The trial Steering Committee decided to terminate recruitment because the performance objectives were reached and the Data Safety Monitoring Board had no safety concerns.

## Results

Between September 2019 and November 2020, 74 patients were included in 9 study centers and treated by 19 different interventionalists with a mean experience of 12 years and performing an average of 68 thrombectomy interventions per year.

All patients who fulfilled inclusion criteria were consecutively included when informed consent could be obtained. There were no patients that were excluded because of anatomic variables.

One patient was a screen failure and therefore excluded from all analyses: This patient was enrolled after confirmation of proximal occlusion on computed tomography angiography and having signed the inform consent. However, at the time of the baseline angiogram the occlusion was recanalized and ANA catheter system was never attempted to be used.

Another patient presented spontaneous recanalization before MT was attempted but after the ANA catheter was introduced through the guiding catheter; this patient was included in the overall population analysis of 73 patients but excluded from the performance analysis. This leaves 72 patients who were included in the ITT analysis. The mITT analysis was performed in 53 patients after excluding 19 roll-in subjects (first patient for each interventionalist). Images were available for core lab evaluation in 69/72 (95.8%) patients. The results are presented following the conservative scenario, which assumes that the missing data from these 3 patients were all performance failures.

### Baseline Characteristics

Mean age was 71.6 (SD 8.9) years old, 34 (46.6%) were women, and 72 (98.6%) presented prestroke mRS score of 0–1. On admission, the median NIHSS score was 14 (interquartile range, 10–19) and median Alberta Stroke Program Early CT Score was 9 (interquartile range, 8–10). Sites of primary occlusion were terminal internal carotid in 10 (13.7%) patients, M1 segment of middle cerebral artery in 37 (50.7%), and M2 segment of middle cerebral artery in 26 (35.6%) patients. Patient characteristics are shown in Table 1.

**Table 1.**
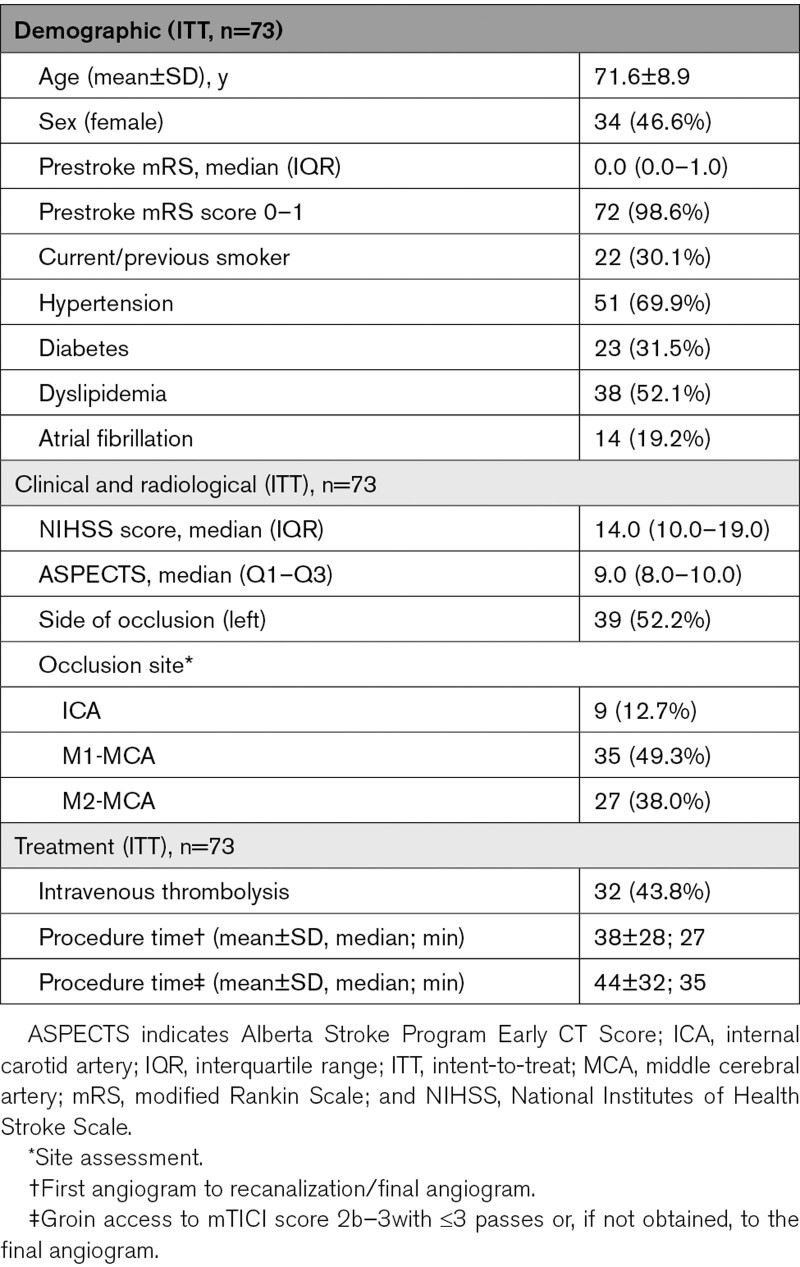
Baseline and Procedure Characteristics

### Primary Performance and Safety Outcomes

Among all patients in whom MT was attempted (the ITT population, n=72), successful reperfusion within 3 passes before rescue therapy was achieved in 60 (83.3%) with a rate of complete reperfusion (mTICI score 2c–3) of 60% (43 patients). In the mITT population (n=53) primary outcome was achieved in 46 (86.8%) patients; 32 patients (60.4%) showed a complete reperfusion. Procedural data of patients evaluated by core lab are shown in Table 2.

**Table 2.**
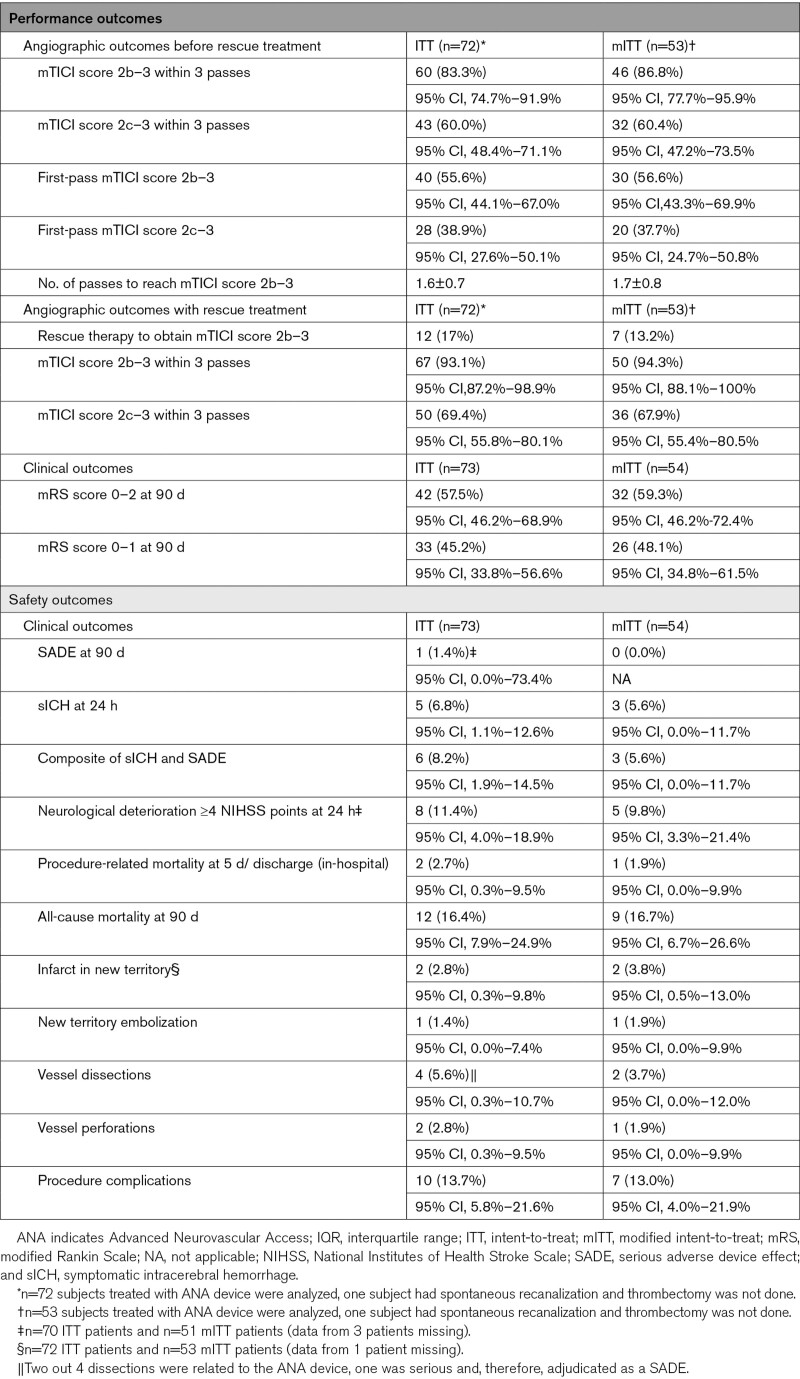
Performance and Safety Outcomes

While noninferiority was confirmed in the ITT (*P*<0.01) and mITT (*P*<0.01) populations, the ANA device also showed superiority in the rate of successful reperfusion in the ITT (*P*=0.02) and mITT (*P*=0.01) populations as compared with the reference population. The noninferiority and superiority analysis tables, and the sensitivity analysis for the patients with missing images at the central core lab are shown in the Supplemental Material.

The rate of SADEs was 1.4% in the ITT population (one patient suffered an arterial dissection); none of the patients from the mITT population presented a SADE. The rate of symptomcatic intracerebral hemorrhage at 24 hours was 6.8% (5/73 patients) and 5.6% (3/54 patients) in the ITT and mITT populations, respectively. The rate of the primary safety end point (the composite of symptomcatic intracerebral hemorrhage and SADE) was 8.2% (6/73 patients) in ITT population, 5.6% (3/54 patients) in mITT (Table 2).

Since the recruitment of patients was not equally distributed among participating centers (the largest enrolling center included 40 patients), we decided to compare outcome measures of patients enrolled in this center with those from all other centers. No significant differences in safety or performance outcome measures were observed (Supplemental Material).

### Secondary Outcomes

Safety outcomes are shown in Table 2. Median procedural time from first angiogram to recanalization, or final angiogram when recanalization was not obtained, was 38 (±28) minutes in the ITT and 36 (±29) in the mITT population. First-pass recanalization rate was 40/72 (56%) with a rate of first-pass complete recanalization of 28/72 (39%); in the mITT population, these rates were 30/53 (57%) and 20/53 (38%). Full distribution of mTICI score is shown in the Supplemental Material. Rescue therapy to obtain a mTICI score 2b–3 was needed in 12/72 (17%) patients. Rate of final mTICI score 2b–3 was 67/72 (93.1%) in ITT population, 50/53 (94.3%) in mITT.

At 90 days, the rate of favorable functional outcome (mRS score 0–2) in the ITT population was 57.5% (42/73) and (59.3% [32/54] in the mITT population) and the rate of excellent functional outcome (mRS score 0–1) 45.2% (33/53) and (48.1% in the mITT population).

Neurological deterioration by 4 or more NIHSS points by 24 hours occurred in 8/73 (11.4%) patients and in-hospital mortality in 2/73 (2.7%) patients in the ITT; 5/53 (9.8%) and 1/53 (1.9%), respectively, in the mITT population. The presence of new vascular infarction was detected in 2/73 (2.7%) patients and procedural complications were reported in 10/73 (13.7%) patients (1 embolization to a new territory, 4 dissections, 2 perforations, and 3 vasospams); 2/53 (3.8%) and 7/53 (13%) in mITT population.

Additional secondary end points are included in the Supplemental Material.

## Discussion

Results of this first-in-man study of the ANA device showed high rates of successful reperfusion within 3 passes without safety concerns. The interim analysis reached the predefined noninferiority primary performance end point derived from the Hermes population.^3^ Moreover, further analyses pointed towards a superior performance when a SR is used in combination with the ANA device.

The observations are in line with the performance shown in preclinical models. Moreover, procedural time was similar to previously published studies of new generation thrombectomy devices or historical series. The minor differences between the ITT and mITT populations together with the mild differences between the results observed in the top recruiting center and the remaining centers suggest a fast learning-curve for the use of the device.

Achieving the highest degree of reperfusion with the minimum attempts,^15^ ideally in a single pass (first-pass effect), has been described as a robust predictor of favorable clinical outcome.^16^ In the SOLONDA study, first-pass effect (mTICI score 2b–3 after one pass) was achieved in 56% of patients and complete recanalization (mTICI score 2c–3) in 39%, which is in a higher range than previously published series.^17^ The combination of local flow arrest with a high catheter-to-vessel diameter may reduce the risk of clot fragmentation and distal embolization while increasing the chances of complete clot retrieval.^6,7^ In the SOLONDA study, the low rate of emboli in new territories (1.4%) suggests a protective effect of the ANA device against this procedural complication.

One of the most frequent reasons of MT failure is related to clot composition. Hard fibrin-rich clots are associated to lower recanalization rates due to reduced clot integration within the SR and a decreased deformability that impairs inclusion into the aspiration catheter.^18,19^ The ANA device, which has the capability to expand up to index artery diameter, showed high rates of recanalization even when hard fibrin-rich clots were used in an in vitro model.^9^

In our study, the high rates of reperfusion were associated to remarkable rates of good (mRS score 0–2: 57.2%) and excellent (mRS score 0–1: 45.2%) functional outcome. Although direct comparisons cannot be done between different studies, these rates remain in the highest ranges of previously published similar series.^20,21^

The safety profile, as evaluated by the independent Data Safety Monitoring Board is in line with results reported in the literature with different thrombectomy devices. The only device-related severe adverse event (1.4%) was an arterial dissection that occurred in the first patient treated by one of the participating interventionalists.

To reduce variability and ease interpretation of the results, the SOLONDA clinical protocol only allowed the use of the Solitaire SR (predominantly used in patients included in the HERMES meta-analysis^3^) in combination with ANA device. However, bench testing showed compatibility of the ANA catheter system with most commercially available SR. Third generation SRs have reported encouraging high rates of reperfusion rates.^20–22^ Due to the complementary nature of the devices, it is possible than the combination of the ANA catheter system with novel SR may lead to even higher recanalization rates.

## Limitations

### Our Study Presents Some Limitations

First of all, the prospective multicentric nature of the study in which all patients were consented and included before treatment initiation avoids reporting bias. Also, the independent core lab assessment and data safety monitoring board minimize potential assessment bias and ensure the highest standards and accuracy of data. Although noninferiority limits were set according to the best available data at the time of study design, recent studies have reported different efficacy results with novel thrombectomy devices, however, not always from prospective, consecutive and centrally evaluated cases.

As this was a single-arm trial, the core lab that evaluated the images was not blinded to the treatment assignment. However, the core lab evaluated the images without knowledge if the pass was done with the ANA device or rescue device.

The effect of receiving intravenous tPA (tissue-type plasminogen activator) just before MT remains unclear among patients with large vessel occlusion; the different rate of intravenous tPA treatment between our study (43.8%), Garcia-Tornel study (43.4%) or the HERMES cohort (83%) may have an incidence on recanalization. The different rates of M2 occlusions between studies may also be considered a limitation.

Also, the impossibility of direct measurement of clot fragmentation is a limitation of this study.

Finally, although no significant differences were observed in the primary outcome there was a numerical difference between the top recruiting center (87.5%) and the others (78.1%) which may have become significant if the study had completed the initially planned sample size.

## Article Information

### Sources of Funding

The SOLONDA trial (Solitaire in Combination With the ANA Catheter System as Manufactured by Anaconda) has been supported by Anaconda Biomed SL.

### Disclosures

Dr Ribo has a consulting agreement with Cerenovus, Medtronic, and Stryker and holds stock at Anaconda. Dr Blasco has consulting agreement with Stryker and receives compensation by Medtronic. Dr Moreu has a consulting agreement with Angionautix, Stryker, and Balt and holds stock at Medtronic. S. Sánchez is employed by Anaconda and holds stock at Anaconda. Dr Liebeskind has a consultant agreement with Cerenovus, Genentech, Medtronic, and Stryker. Dr Andersson has a consultant agreement with Johnson & Johnson, Neuravi, Rapid Medical, and Stryker. Dr Cognard has a consultant agreement with Anaconda, Johnson & Johnson, MicroVention, and Stryker. Dr Nogueira has a consultant agreement with Anaconda, Biogen, Brainomix, Cerenovus, Ceretrieve, Corindus, Genentech, Imperative Care, Medtronic, NeuroVasc, Perfuze, Phenox, Prolong, Stryker, Vesalio, and Viz-AI and hols stock at Brainomix, Ceretrieve, Corindus, Perfuze, Vesalio and Viz-AI. Dr Tomasello has a consultant agreement with Balt, Medtronic, Perflow, and Stryker. The other authors report no conflicts.

### Supplemental Material

Tables S1–S7

CONSORT 2010 flow diagram

## Supplementary Material


