# Supplementary material for: Clinical Results of the Advanced Neurovascular Access Catheter System Combined With a Stent Retriever in Acute Ischemic Stroke (SOLONDA)
Source: Stroke. 2022 Apr 1;53(7):2211–9. doi: 10.1161/STROKEAHA.121.037577 (PMC9232250; doi:10.1161/STROKEAHA.121.037577)
Supplement: Supplementary file 2 [file str-53-2211-s002.pdf]

SUPPLEMENTAL MATERIAL

Table S1. Exclusion and inclusion criteria

**Inclusion Criteria:**	Clinical<br>1. Age ≥18 and ≤85 years.<br>2. Informed consent obtained from subject or acceptable subject surrogate (i.e. next of kin, or legal representative).<br>3. A new focal disabling neurologic deficit consistent with acute cerebral ischemia.<br>4. Baseline NIHSS obtained prior to procedure ≥ 8 points and ≤ 25 points.<br>5. Pre-ictal mRS score of 0 or 1.<br>6. Treatable as soon as possible and at least within 8 h of symptom onset, defined as point in time when the subject was last seen well (at baseline). (Treatment start is defined as groin puncture.)<br>7. Subjects for whom intravenous (IV) tissue plasminogen activator (t-PA) is indicated and who are available for treatment, are treated with IV t-PA. For such patients, IV t-PA should be administered as recommended by the American Heart Association/American Stroke Association (AHA/ASA) Guidelines for the early management of patients with AIS.<br>8. IV t-PA, if used, is initiated as soon as possible and within 3 h of stroke onset (onset time is defined as the last time when the patient was witnessed to be well at baseline), with investigator verification that the subject has received/is receiving the correct IV t-PA dose for the estimated weight.<br>Neuro Imaging<br>9. Occlusion (TICI 0 or TICI 1 flow), of the terminal internal carotid artery, M1 or M2 segments of the middle cerebral artery, suitable for mechanical embolectomy, confirmed on conventional angiography.<br>10. The following imaging criteria should also be met:<br>a) MRI criterion: volume of diffusion restriction visually assessed ≤50 mL.<br>OR<br>b) CT criterion: Alberta Stroke program early CT score (ASPECTS) 6 to 10 on baseline CT or CT-Angiography (CTA)-source images, or, volume of significantly lowered Cerebral Blood Volume (CBV) ≤50 mL.<br>11. The subject is indicated for neurothrombectomy treatment by the Interventionalist.
**Exclusion Criteria:**	Clinical<br>1. Pre-stroke functional disability (mRS score >1).<br>2. Initially treated with a different thrombectomy device.

3. Subject has suffered a stroke in the past 1 year.
4. Occlusion (TICI 0 or TICI 1 flow) of the basilar or vertebral arteries
5. The subject presents with an NIHSS score <8 or >25.
6. Clinical symptoms suggestive of bilateral stroke or stroke in multiple territories.
7. Known hemorrhagic diathesis, coagulation factor deficiency, or oral anticoagulant therapy with INR >3.0.
8. Baseline platelet count <50,000/µL.
9. Baseline blood glucose of <50 mg/dL or >400 mg/dL.
10. Severe, sustained hypertension (systolic blood pressure >185 mmHg or diastolic blood pressure >110 mmHg).

NOTE: If the blood pressure can be successfully reduced and maintained at an acceptable level using European Stroke Organization (ESO) guidelines recommended medication (including IV antihypertensive drips), the patient can be enrolled.

11. Serious, advanced, or terminal illness with anticipated life expectancy of less than 1 year.
12. Subjects with identifiable intracranial tumors.
13. History of life-threatening allergy (more than rash) to contrast medium.
14. Known nickel allergy at time of treatment.
15. Known renal insufficiency with creatinine ≥3 mg/dL or Glomerular Filtration Rate (GFR) <30 mL/min.
16. Cerebral vasculitis.
17. Evidence of active systemic infection.
18. Known current use of cocaine at time of treatment.
19. Woman of childbearing potential who is known to be pregnant, and/or lactating, or who has a positive pregnancy test on admission.
20. Patient participating in a study involving an investigational drug or device that would impact this study.
21. Patients that are unlikely to be available for a 90-day follow-up (e.g. no fixed home address, visitor from overseas).

<u>Neuro Imaging</u>

22. Hypodensity on CT or restricted diffusion amounting to an Alberta Stroke Program Early CT (ASPECTS) score of <6 on CT or <5 on diffusion weighted (DW) MRI.
23. CT or MRI evidence of hemorrhage (the presence of microbleeds is

	allowed).<br>24. Angiographic evidence of carotid dissection, high grade stenosis or vasculitis.<br>25. Significant mass effect with midline shift.<br>26. Evidence of complete occlusion, high grade stenosis or arterial dissection in the extracranial or petrous segment of the internal carotid artery.<br>27. Subjects with known or suspected underlying intracranial atherosclerotic lesions responsible for the target occlusion.<br>28. Subjects with occlusions in multiple vascular territories (e.g., bilateral anterior circulation, or anterior/posterior circulation).<br>29. Evidence of intracranial tumor.<br>30. Suspicion of aortic dissection presumed septic embolus, or suspicion of bacterial endocarditis.<br>31. Severe arterial tortuosity avoiding stable positioning of the guide catheter in the petrous segment (C2) of Internal Carotid Artery (ICA)

CONSORT 2010 Flow Diagram

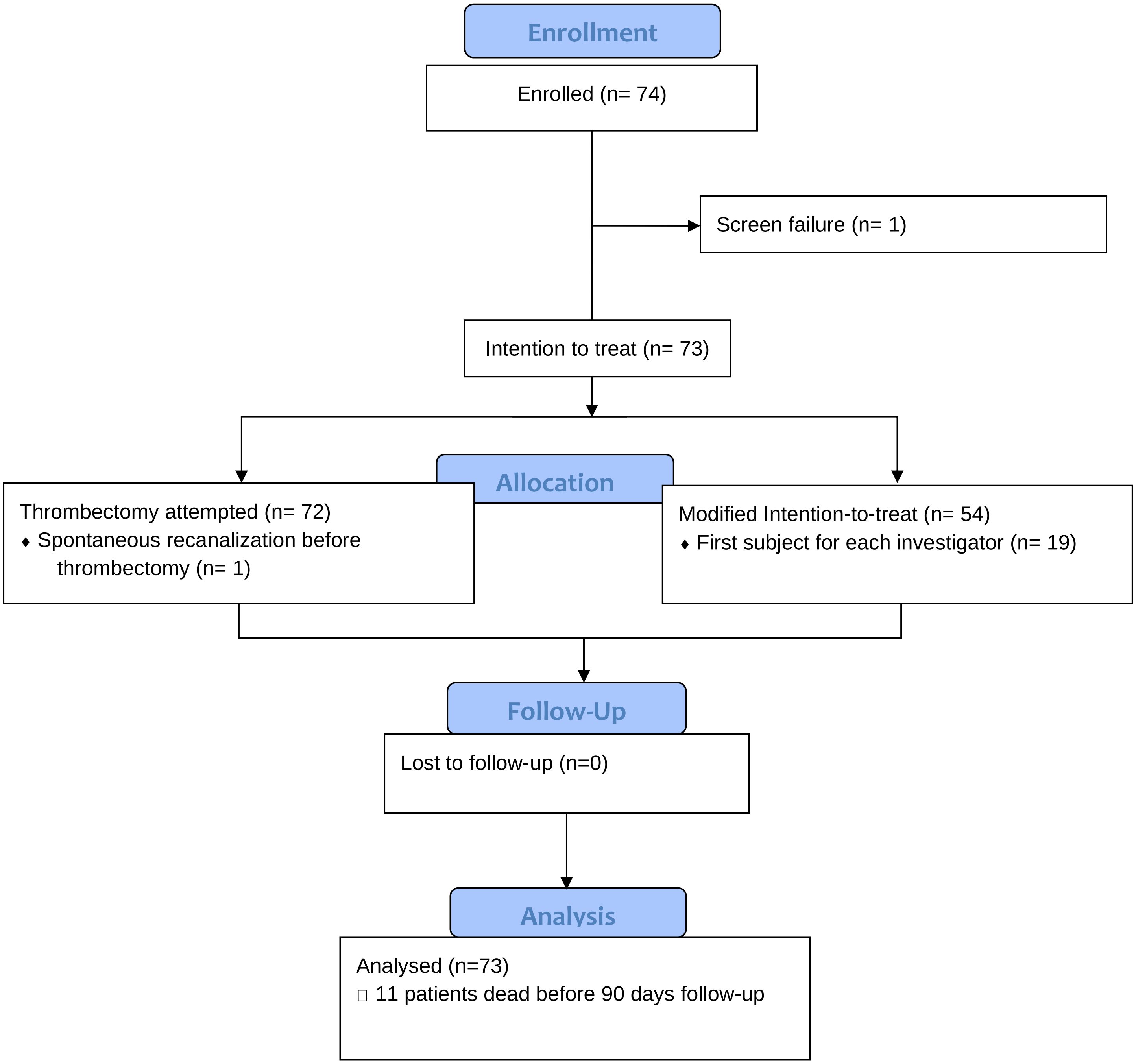

Table S2. Inferiority and superiority analysis of the primary performance endpoint

Primary performance endpoint (Data Set 2) – mTICI in the ITT population (non-inferiority):

Data Set 2 (Core Lab data + eCRF data for missing Core Lab data)		Intention-to-Treat (ITT) (N=73)	P-value
Primary Performance Endpoint (PPE) (mTICI score ≥2b)	N	72	0.0006
	No	12 (16.7%)	
	95% CI (No)	[8.1% - 25.3%]	
	Yes	60 (83.3%)	
	95% CI (Yes)	[74.7% - 91.9%]	
	Missing	1	

Primary performance endpoint (Data Set 2) – mTICI in the mITT population (non-inferiority):

Data Set 2 (Core Lab data + eCRF data for missing Core Lab data)		Modified Intention-to-Treat (mITT) (N=54)	P-value
Primary Performance Endpoint (PPE) (mTICI score ≥2b)	N	53	<.0001
	No	7 (13.2%)	
	95% CI (No)	[4.1% - 22.3%]	
	Yes	46 (86.8%)	
	95% CI (Yes)	[77.7% - 95.9%]	
	Missing	1	

Primary performance endpoint (Data Set 2) – mTICI in the ITT population (superiority)

Data Set 2 (Core Lab data + eCRF data for missing Core Lab data)		Intention-to-Treat (ITT) (N=73)	P-value
Primary Performance Endpoint (PPE) (mTICI score ≥2b)	N	72	0.0211
	No	12 (16.7%)	
	95% CI (No)	[8.1% - 25.3%]	
	Yes	60 (83.3%)	
	95% CI (Yes)	[74.7% - 91.9%]	
	Missing	1	

Primary performance endpoint (Data Set 2) – mTICI in the mITT population (superiority)

Data Set 2 (Core Lab data + eCRF data for missing Core Lab data)		Modified Intention-to-Treat (mITT) (N=54)	P-value
Primary Performance Endpoint (PPE) (mTICI score ≥2b)	N	53	0.0113
	No	7 (13.2%)	
	95% CI (No)	[4.1% - 22.3%]	
	Yes	46 (86.8%)	
	95% CI (Yes)	[77.7% - 95.9%]	
	Missing	1	

Table S3. Sensitivity analysis of the primary performance endpoint

Primary performance endpoint (Data Set 2) – mTICI in the ITT population – Sensitivity analysis – Modality 1 (non-inferiority)

Data Set 2 (Core Lab data + eCRF data for missing Core Lab data)		Intention-to-Treat (ITT) (N=73)	P-value
Primary Performance Endpoint (PPE) (mTICI score ≥2b)	N	73	0.0016
	No	13 (17.8%)	
	95% CI (No)	[9.0% - 26.6%]	
	Yes	60 (82.2%)	
	95% CI (Yes)	[73.4% - 91.0%]	
	Missing	0	

Primary performance endpoint (Data Set 2) – mTICI in the ITT population – Sensitivity analysis – Modality 2 (non-inferiority)

Data Set 2 (Core Lab data + eCRF data for missing Core Lab data)		Intention-to-Treat (ITT) (N=73)	P-value
Primary Performance Endpoint (PPE) (mTICI score ≥2b)	N	73	0.0004
	No	12 (16.4%)	
	95% CI (No)	[7.9% - 24.9%]	
	Yes	61 (83.6%)	
	95% CI (Yes)	[75.1% - 92.1%]	
	Missing	0	

Primary performance endpoint (Data Set 2) – mTICI in the ITT population – Sensitivity analysis – Modality 1 (superiority)

Data Set 2 (Core Lab data + eCRF data for missing Core Lab data)		Intention-to-Treat (ITT) (N=73)	P-value
Primary Performance Endpoint (PPE) (mTICI score ≥2b)	N	73	0.0351
	No	13 (17.8%)	
	95% CI (No)	[9.0% - 26.6%]	
	Yes	60 (82.2%)	
	95% CI (Yes)	[73.4% - 91.0%]	

Data Set 2 (Core Lab data + eCRF data for missing Core Lab data)		Intention-to-Treat (ITT) (N=73)	P-value
	Missing	0	

Primary performance endpoint (Data Set 2) – mTICI in the ITT population – Sensitivity analysis – Modality 2 (superiority)

Data Set 2 (Core Lab data + eCRF data for missing Core Lab data)		Intention-to-Treat (ITT) (N=73)	P-value
Primary Performance Endpoint (PPE) (mTICI score ≥2b)	N	73	0.0180
	No	12 (16.4%)	
	95% CI (No)	[7.9% - 24.9%]	
	Yes	61 (83.6%)	
	95% CI (Yes)	[75.1% - 92.1%]	
	Missing	0	

Table S4. Assessment of mTICI in the ITT population per site

Data Set 2 (Core Lab data + eCRF data for missing Core Lab data)		Group of sites		
		Site 11 (N=40)	Sites 12-19 (N=33)	P-value
Primary Performance Endpoint (PPE) (mTICI score ≥2b)	N	40	32	
	No	5 (12.5%)	7 (21.9%)	0.289
	95% CI (No)	[4.2% - 26.8%]	[7.6% - 36.2%]	(Chi²)
	Yes	35 (87.5%)	25 (78.1%)	
	95% CI (Yes)	[73.2% - 95.8%]	[63.8% - 92.4%]	
	Missing	0	1	

Table S5. Performance outcomes

**Angiographic outcomes without rescue treatment**	**Intention-to-Treat (ITT) (n=73)***
mTICI 0	8/72 (11.1%)
mTICI 1	1/72 (1.4%)
mTICI 2a	3/72 (4.2%)
mTICI 2b	17/72 (23.6%)
mTICI 2c	13/72 (18.1%)
mTICI 3	30/72 (41.7%)
**Angiographic outcomes with rescue treatment**	**Intention-to-Treat (ITT) (n=73)***
mTICI 0	8/72 (11.1%)
mTICI 1	1/72 (1.4%)
mTICI 2a	3/72 (4.2%)
mTICI 2b	17/72 (23.6%)
mTICI 2c	13/72 (18.1%)
mTICI 3	30/72 (41.7%)
**Assessment of navigation**	**Intention-to-Treat (ITT) (n=73)**
Navigation of the ANA System to pass the bulb of the ICA	66/67 (98.5%)**
Combination of ANA System with the stent retriever (navigation and deployment of the stent retriever to attempt neurothrombectomy)	70/70 (100%)***

*1 ITT subject was finally not treated with mechanical thrombectomy (spontaneous reperfusion)
**data not available for 5 subjects
*** in 2 subjects the stent retriever was not introduced because the ANA System was removed and changed for a rescue device

	**Mean**	**S.D.**	**Median**	**Min, Max**	**Q1-Q3**	**95% CI**
**Procedure time (minutes) Intention-to-Treat (ITT) (n=73)**	44.2	31.9	35.0	7.0, 131.0	21.0 - 56.0	[36.6; 51.8]
**Procedure time (minutes) mITT (n=54)**	41.5	32.7	30.0	7.0, 131.0	19.0 - 46.0	[32.5; 50.5]
**Time to treat (minutes) Intention-to-Treat (ITT) (n=73)**	30.3	18.5	25.0	4.0, 97.0	17.0 - 39.0	[25.9; 34.7]

	Mean	S.D.	Median	Min, Max	Q1-Q3	95% CI
**Time to treat (minutes) mITT (n=54)**	27.2	14.8	23.5	4.0, 84.0	17.0 - 34.5	[23.0; 31.3]

**Clinical outcomes**	**Intention-to-Treat (ITT) (n=73)**	**Modified Intention-to-Treat (mITT) (n=54)**
NIHSS at 5 days (median, IQR)	2.5 (1.0-6.0)	2.0 (1.0-5.5)
mRS at 90 days (median, IQR)	2.0 (1.0-4.0)	2.0 (1.0-4.0)

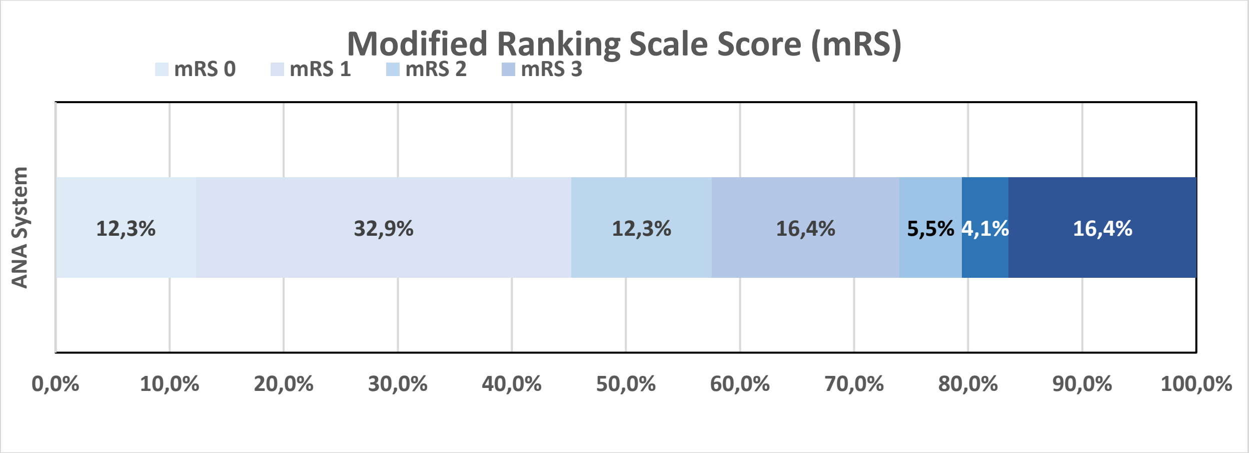

Table S6. Safety outcomes

Clinical outcomes	Intention-to-Treat (ITT) (n=73)	Modified Intention-to-Treat (mITT) (n=54)
Asymptomatic intracerebral hemorrhage at 24h	24 (33.8%)	18 (34.6%)

Intracerebral hemorrhage at 24h classification (Core Lab)*	Intention-to-Treat (ITT) (N=73)
N	29
HI-1	14 (48.3%)
HI-1 SAH	1 (3.4%)
HI-2	1 (3.4%)
PH-2 SAH	1 (3.4%)
SAH	11 (37.9%)
SAH PH-2	1 (3.4%)

* HI-1 Scattered small petechiae, no mass effect; HI-2 Confluent petechiae, no mass effect; PH1 Hematoma within infarcted tissue, occupying <30%, no substantive mass effect; PH-2 Hematoma occupying 30% or more of the infarcted tissue, with obvious mass effect; SAH Subarachnoid hemorrhage.

Adverse events at 90 days	Intention-to-Treat (ITT) (N=73)
ALL	197
Infection	7
Intracranial hemorrhage*	29
Vessel dissection*	4
Vessel perforation*	2
Infarct	3
Vasospasm	3
Distal embolization*	22
Embolization to new vascular territory*	1
Device malfunction, damage and/or failure	2
Pain	1
Stroke / reoccurring stroke	1

Adverse events at 90 days	Intention-to-Treat (ITT) (N=73)
Neurological deficit	1
Other	122

* Core Lab assessment

Serious adverse events at 90 days	Intention-to-Treat (ITT) (N=73)
ALL	39
Intracranial hemorrhage*	8
Vessel dissection	3
Vessel perforation*	2
Infection	2
Embolization to new vascular territory*	0
Infarct	1
Stroke / reoccurring stroke	1
Other	20

* Core Lab assessment

Table S7. Performance and safety outcomes in the Solonda study and other published studies

Performance endpoints	SOLONDA (n=73, ITT)	Published Studies											
		HERMES (n=634)[1]	ARISE (n=227)[2]	TREVO registry (TRACK) (n=629)[3]	REVASCAT (n=103)[4]	SWIFT PRIME (n=98)[5]	MR CLEAN Registry (n=528)[6]	EXTEND IA (n=35)[7]	ESCAPE (n=165)[8]	SWIFT (n=58)[9]	Sudden Rec (n=609)[10]	STRATIS registry (n=445)[11]	SEER (n=441)[12]
**Device**	**ANA + Solitaire**	**Multiple**	**Embotrap**	**Trevo**	**Multiple**	**Solitaire**	**Multiple**	**Solitaire**	**Solitaire**	**Solitaire**	**Multiple**	**Multiple**	**Solitaire**
Successful revascularization mTICI 2b-3 [%] 3 passes	83.3%		80.2%						72.4%	69.0%			
Successful revascularization mTICI 2c-3 [%] 3 passes	59.7%		64.8%										
First pass mTICI 2b-3 [%]	55.6%		51.5%									66.4%	
First pass mTICI 2c-3 [%]	38.9%		40.1%									47.9%	
Number of passes successful revascularization	1.6 ± 0.7		1.9 ± 1.2										
Successful revascularization mTICI 2b-3 [%] Final	93.1%	71.1%	92.5%	80.3%	66.0%	88.0%	75.0%	86.0%	76.0%	89.0%	83.6%	89.0%	71.1%

1 *Goyal M, Menon BK, van Zwam WH, Dippel DW, Mitchell PJ, Demchuk AM, et al; HERMES collaborators. Endovascular thrombectomy after large-vessel ischaemic stroke: a meta-analysis of individual patient data from five randomized trials (HERMES metanalysis). Lancet. 2016;387:1723–1731.*

2 *Zaidat OO, Bozorgchami H, Ribo M, et al. Primary Results of the Multicenter ARISE II Study (Analysis of Revascularization in Ischemic Stroke with EmboTrap). Stroke. 2018;49(5):1107-1115.*

3 *Zaidat OO, Castonguay AC, Nogueira RG, et al.TREVO stent-retriever mechanical thrombectomy for acute ischemic stroke secondary to large vessel occlusion registry. J Neurointerv Surg. 2018 Jun;10(6):516-524.*

4 *Jovin TG, Chamorro A, Cobo E, de Miquel MA, et al.; REVASCAT Trial Investigators. Thrombectomy within 8 hours after symptom onset in ischemic stroke. N Engl J Med. 2015 Jun 11;372(24):2296-306.*

5 *Saver JL, Goyal M, Bonafe A, et al., SWIFT PRIME Investigators. Stent retriever thrombectomy after intravenous t-PA vs. t-PA alone in stroke. N Engl J Med 2015. 372:2285–2295.*

6 *Berkhemer OA, Fransen PS, Beumer D, et al. MR CLEAN Investigators. A randomized trial of intraarterial treatment for acute ischemic stroke. N Engl J Med 2015; 372: 11–20.*

7 *Campbell BC, Mitchell PJ, Kleinig TJ, et al. EXTEND-IA Investigators. Endovascular therapy for ischemic stroke with perfusion-imaging. N Engl J Med. 2015 Mar 12;372(11):1009-18.*

8 *Goyal M, Demchuk AM, Menon BK, et al. Randomized assessment of rapid endovascular treatment of ischemic stroke. N Engl J Med. 2015 Mar 12;372(11):1019-30.*

9 *Saver JL, Jahan R, Levy EI, et al. Solitaire flow restoration device versus the Merci Retriever in patients with acute ischaemic stroke (SWIFT): a randomised, parallel-group, non-inferiority trial. Lancet 2012; 380:1241–9.*

10 *Garcia-Tornel A, Rubiera M, Requena M, et al. Sudden recanalization: a game-changing factor in endovascular treatment of large vessel occlusion strokes. Stroke. 2020;51(4):1313–1316.*

11 *Zaidat OO, Mueller-Kronast NH, Hassan AE, et al. Impact of Balloon Guide Catheter Use on Clinical and Angiographic Outcomes in the STRATIS Stroke Thrombectomy Registry. Stroke 2019;50:697-704.*

12 *Campbell Bc, Hill Md, Rubiera M, et al. Safety and efficacy of solitaire stent thrombectomy: individual patient data meta-analysis of randomized trials. Stroke. 2016;47(3):798–806.*

Performance endpoints	SOLONDA (n=73, ITT)	Published Studies											
		HERMES (n=634)[1]	ARISE (n=227)[2]	TREVO registry (TRACK) (n=629)[3]	REVASCAT (n=103)[4]	SWIFT PRIME (n=98)[5]	MR CLEAN Registry (n=528)[6]	EXTEND IA (n=35)[7]	ESCAPE (n=165)[8]	SWIFT (n=58)[9]	Sudden Rec (n=609)[10]	STRATIS registry (n=445)[11]	SEER (n=441)[12]
Successful revascularization mTICI 2c-3 [%] Final	69.4%	31.4%	75.8%				52.0%						
Successful revascularization mTICI 3 [%] Final	48.6%	8.6%	43.6%	44.5%			41.0%				45.6%		32.9%
Procedure time (min)	44.2 ± 31.9		36	67	75			43	30			42.4 ± 25.7	
mRS 0-2 90 days [%]	57.5%	46.0%	67.3%	48.0%	43.7%		61.0%	71.0%	53.0%	58.0%	44.8%	61.0%	54.0%
sICH 24h [%]	6.8%	4.4%	5.3%	7.1%	4.9%	0.0%	4.0%	0.0%	3.6%	2.0%		4.1%	2.8%
SADE 90 days [%]	1.4%		0.0%							9.0%			
All cause-mortality 90 days [%]	16.4%	15.3%	9.0%	19.8%	18.4%	9.0%	23.0%	8.6%	10.4%	17.0%		14.1%	12.0%
Embolization New Territory [%]	1.4%		6.6%	4.5%	4.9%			6.0%				4.2%	
Vessel Dissection [%]	5.6%		1.8%		3.9%							1.1%	
Vessel Perforation [%]	2.8%		1.8%		4.9%			2.8%				1.2%	
Vasospasm [%]	4.2%				3.9%							7.4%	
